# Near-infrared spectroscopy for assessing tissue oxygenation and microvascular reactivity in critically ill patients: a prospective observational study

**DOI:** 10.1186/s13054-016-1500-5

**Published:** 2016-10-01

**Authors:** Abele Donati, Elisa Damiani, Roberta Domizi, Claudia Scorcella, Andrea Carsetti, Stefania Tondi, Valentina Monaldi, Erica Adrario, Rocco Romano, Paolo Pelaia, Mervyn Singer

**Affiliations:** 1 Anesthesia and Intensive Care Unit, Department of Biomedical Sciences and Public Health, Università Politecnica delle Marche, Ancona, Italy; 2 Bloomsbury Institute of Intensive Care Medicine, Division of Medicine, University College London, London, UK

**Keywords:** Tissue oxygenation, Microcirculation, Near-infrared spectroscopy, Vascular occlusion test, Critical illness

## Abstract

**Background:**

Impaired microcirculatory perfusion and tissue oxygenation during critical illness are associated with adverse outcome. The aim of this study was to detect alterations in tissue oxygenation or microvascular reactivity and their ability to predict outcome in critically ill patients using thenar near-infrared spectroscopy (NIRS) with a vascular occlusion test (VOT).

**Methods:**

Prospective observational study in critically ill adults admitted to a 12-bed intensive care unit (ICU) of a University Hospital. NIRS with a VOT (using a 40 % tissue oxygen saturation (StO_2_) target) was applied daily until discharge from the ICU or death. A group of healthy volunteers were evaluated in a single session. During occlusion, StO_2_ downslope was measured separately for the first (downslope 1) and last part (downslope 2) of the desaturation curve. The difference between downslope 2 and 1 was calculated (delta-downslope). The upslope and area of the hyperaemic phase (receive operating characteristic (ROC) area under the curve (AUC) of StO_2_) were calculated, reflecting microvascular reactivity. Outcomes were ICU and 90-day mortality.

**Results:**

Patients (*n* = 89) had altered downslopes and upslopes compared to healthy volunteers (*n* = 27). Mean delta-downslope was higher in ICU non-survivors (2.8 (0.4, 3.8) %/minute versus 0.4 (−0.8, 1.8) in survivors, *p* = 0.004) and discriminated 90-day mortality (ROC AUC 0.72 (95 % confidence interval 0.59, 0.84)). ICU non-survivors had lower mean upslope (141 (75, 193) %/minute versus 185 (143, 217) in survivors, *p* = 0.016) and AUC StO_2_ (7.9 (4.3, 12.6) versus 14.5 (11.2, 21.3), *p* = 0.001). Upslope and AUC StO_2_ on admission were significant although weak predictors of 90-day mortality (ROC AUC = 0.68 (0.54, 0.82) and 0.70 (0.58, 0.82), respectively). AUC StO_2_ ≤ 6.65 (1st quartile) on admission was independently associated with higher 90-day mortality (hazard ratio 7.964 (95 % CI 2.211, 28.686)). The lowest upslope in the ICU was independently associated with survival after ICU discharge (odds ratio 0.970 (95 % CI 0.945, 0.996)).

**Conclusions:**

In critically ill patients, NIRS with a VOT enables identification of alterations in tissue oxygen extraction capacity and microvascular reactivity that can predict mortality.

**Trial registration:**

NCT02649088, www.clinicaltrials.gov, date of registration 23rd December 2015, retrospectively registered.

**Electronic supplementary material:**

The online version of this article (doi:10.1186/s13054-016-1500-5) contains supplementary material, which is available to authorized users.

## Background

Imbalances between oxygen (O_2_) delivery and demand during critical illness may result in tissue hypoxia and lead to organ dysfunction and death. The first goal of treatment is to optimize tissue perfusion and O_2_ supply. Nonetheless, commonly used hemodynamic targets (mean arterial pressure (MAP) and cardiac output) or markers of global oxygenation such as central venous O_2_ saturation (SvO_2_) or arterial lactate, are not always specific or early predictors of organ perfusion [1]. Increasing evidence suggests a potential dissociation between microcirculation and macro-haemodynamics in the critically ill [2–4]: microcirculatory hypoperfusion may thus persist despite normalization of global haemodynamic parameters [5].

Near-infrared spectroscopy (NIRS) was introduced some decades ago as a non-invasive tool for measuring oxygenation in the muscle and other tissues, although the thenar eminence is the most widely tested site [6]. Several studies report an association between low thenar tissue O_2_ saturation (StO_2_) and poor outcome, especially during sepsis [7–10]. In conjunction with a vascular occlusion test (VOT), NIRS allows analysis of changes in StO_2_ during a brief ischaemic challenge, providing dynamic parameters of tissue O_2_ extraction and microvascular reactivity [10]. Slower StO_2_ recovery during the reperfusion phase is an independent predictor of mortality in patients with sepsis [9].

By using NIRS with a VOT daily in a cohort of patients admitted to our intensive care unit (ICU), we aimed to confirm the relationship between static or dynamic NIRS-derived variables and outcome and further characterize their prognostic value during critical illness.

## Methods

This is a secondary analysis of the microcirculatory daily monitoring in ICU (MICRODAIMON-ICU) study (NCT02649088, www.clinicaltrails.gov), a single-centre prospective observational study in a population of 100 critically ill patients, with the primary goal of evaluating the relationship between changes in the sublingual microcirculation during the ICU stay and outcome. This study was performed between April and December 2013 in a 12-bed ICU of Azienda Ospedaliera Universitaria “Ospedali Riuniti” in Ancona, Italy. At the time of enrolment, our ICU was divided into three sub-sections (General, Trauma and Respiratory ICU) with four beds in each. All consecutive adult (≥18 years old) patients admitted to each section during three trimesters (General: April–June 2013; Trauma: July–September 2013; Respiratory: October–December 2013) were included. Exclusion criteria were recent maxillo-facial surgery/trauma and pregnancy.

Every day until ICU discharge or death of the patient, the patients underwent microcirculatory assessment through sublingual videomicroscopy and NIRS monitoring. A group of healthy volunteers were studied in a single session as controls. The study was approved by our local ethical committee of Azienda Ospedaliera Universitaria “Ospedali Riuniti” of Ancona, Italy. Written informed consent was obtained from all patients or their next of kin.

### NIRS monitoring

An InSpectra StO_2_ Tissue Oxygenation Monitor (model 650; Hutchinson Technology, Hutchinson, MN, USA) was used to measure StO_2_ at baseline and during a VOT with a 15-mm-spaced probe applied on the thenar eminence, as described previously [11, 12]. After a 3-minute period of StO_2_ signal stabilization, arterial inflow was arrested by inflation of a sphygmomanometer cuff to 50 mmHg above the systolic arterial pressure. The cuff was kept inflated until StO_2_ decreased to 40 % and was then released [13]. StO_2_ was continuously recorded during the reperfusion phase until stabilization.

NIRS-derived parameters were calculated using a software package (version 3.03 InSpectra Analysis Program; Hutchinson Technology Inc.). StO_2_ and tissue haemoglobin index (THI) [14] were calculated at baseline. The StO_2_ downslope (%/minute) is generally calculated from the regression line of the first part of StO_2_ decay after occlusion, providing an index of tissue O_2_ extraction rate [15]. However, we noted that the desaturation slope may vary during the ischaemic phase of the VOT, becoming more steep or less steep before reaching the 40 % StO_2_ threshold. In order to explore the meaning of this variation in the desaturation slope, the inflection point was visually identified and the downslope was calculated separately for the first and the last part of the desaturation curve (downslope 1 and downslope 2, respectively). Whenever a change in the slope was not observed, the desaturation curve was divided into two halves for the calculation of the two downslope values. The delta-downslope was then calculated as the difference between the last and the first part of the desaturation slope (downslope 2 – downslope 1), so that a positive value indicated a flattening in the second part of the slope (slower StO_2_ decay). The StO_2_ upslope (%/minute) and the area under the curve of the hyperaemic response (AUC StO_2_) were calculated as indices of microvascular reactivity [13].

### Clinical parameters and outcomes

For all patients we recorded age, gender, reason for ICU admission, comorbidities and acute physiology and chronic evaluation (APACHE) II score on admission. The sequential organ failure assessment (SOFA) score, presence of sepsis (as defined according to standard criteria [16]), main clinical and laboratory parameters and arterial and venous blood gas analyses were recorded every day simultaneously with NIRS measurements. Outcomes of interest were ICU mortality and 90-day mortality.

### Statistical analysis

Statistical analysis was performed using IBM SPSS (version 19), GraphPad Prism version 5 (GraphPad software, La Jolla, CA, USA) and MedCalc version 12.5 (MedCalc software, Ostend, Belgium). Normality of distribution was checked using the Kolmogorov-Smirnov test. Continuous variables were expressed as mean ± standard deviation or median (25th–75th percentile), as appropriate. Student’s *t* test or the Mann–Whitney *U* test was used to compare continuous variables between two groups. Nominal variables were compared across groups using the chi-square test. The Kruskal-Wallis test with Dunn’s test for multiple comparisons was used to compare data between more than two groups. The area under the receiver operating characteristic (ROC) curve was calculated to evaluate the predictive value of the variables for 90-day mortality. The Logrank Mantel Cox test and multivariable Cox regression were used to evaluate differences in survival between patients stratified based on quartiles of NIRS variables. Multivariate binary logistic regression was performed to evaluate the independent association between NIRS-derived variables and outcome. A *p* value <0.05 was used to indicate statistical significance.

## Results

A total of 27 healthy volunteers (age 30 (28–52) years; 10 male, 17 female) and 89 patients (age 66 (45–74) years; 62 male, 27 female) were studied. NIRS monitoring was not performed in 11 of the total cohort of 100 patients because of bilateral upper limb fractures, patient’s refusal to participate or unavailability of the NIRS device at the time of enrolment. Mean length of stay in the ICU was 8 [(4–15) days. Eighteen patients (20 %) died in the ICU, while 90-day mortality was 31 % (28 patients out of 89). On admission, ICU non-survivors had higher APACHE II and SOFA scores, higher heart rate (HR), lower MAP, higher lactate and had higher probability of receiving vasopressors (Table 1). NIRS-derived variables for ICU survivors and non-survivors are shown in Table 2.

**Table 1:** Description of the patients on admission to the ICU: comparison between ICU survivors and non-survivors

	ICU survivors (*n* = 71)	ICU non-survivors (*n* = 18)	*P*
Age (years)	59 ± 18	67 ± 18	0.080
Gender (*n*, male; female)	49; 22	13; 5	0.518
APACHE II score	15 ± 7	23 ± 5	<0.001
SOFA score	6 ± 3	11 ± 5	<0.001
Admission diagnosis (*n*)			0.131
Trauma (36)	33	3	
Neurologic (20)	17	3	
Respiratory (8)	6	2	
Sepsis (8)	5	3	
Surgery^a^ (7)	4	3	
Cardiac (3)	2	1	
Other (7)	4	3	
Comorbidities (*n*)			0.362
None (34)	30	4	
Arterial hypertension (27)	20	7	
Chronic vascular disease (17)	11	6	
Cardiac disease (24)	16	8	
Chronic respiratory disease (15)	8	7	
Diabetes mellitus (14)	12	2	
Obesity (13)	9	4	
Cancer (8)	5	3	
Hypercholesterolaemia (6)	4	2	
Chronic renal failure (6)	4	2	
ICU length of stay	9 (5–15)	3 (2–11)	0.006
Heart rate (bpm)	80 ± 22	93 ± 28	0.036
Mean arterial pressure (mmHg)	87 (74–95)	74 (56–87)	0.046
Hb (g/dL)	11 ± 1.5	11 ± 2.3	0.959
Arterial lactate (mmol/L)	1.3 (0.9–1.8)	3.1 (1.5–5.6)	<0.001
Any vaspressor			0.006
Yes	34	15	
No	37	3	

Results are presented as mean ± SD or median (1st-3rd quartile). ^a^Three high-risk scheduled surgical operations (two pulmonary lobectomies, one partial pancreatectomy complicated by intra-operative bleeding), four emergency interventions (three ruptured abdominal aortic aneurysms, one fasciotomy for soft tissue infection). *APACHE* acute physiology and chronic evaluation, *SOFA* sequential organ failure assessment

**Table 2:** Near-infrared spectroscopy-derived parameters in ICU survivors and non-survivors

	ICU survivors (*n* = 71)	ICU non-survivors (*n* = 18)	*P*
First measurement			
StO_2_ (%)	78 (70–82)	81 (75–85)	0.176
Downslope 1 (%/minute)	−8.1 (−9.6 - −6.7)	−7.9 (−10 - −6.2)	0.764
Downslope 2 (%/minute)	−7.7 (−10.5 - −5.6)	−6.7 (−10.5 - −3.4)	0.200
Delta-downslope (%/minute)	0.1 (−1.2 - 1.7)	2.1 (−0.5 - 3.6)	0.050
Upslope (%/minute)	150 (114–206)	103 (44–224)	0.202
AUC StO_2_	14.6 (9.2-26.8)	4.3 (0.5-16.6)	0.002
Tissue haemoglobin index	10.9 (8.4-12.9)	10.9 (8.4-13.7)	0.612
Mean values			
StO_2_ (%)	80 (76–84)	79 (73–85)	0.567
Downslope 1 (%/minute)	−8.8 (−10.3 - −7.6)	−8.7 (−10.5 - −7.9)	0.927
Downslope 2 (%/minute)	−9.0 (−10 - −7.2)	−6.5 (−8.6 - −5.4)	0.010
Delta-downslope (%/minute)	0.4 (−0.8 - 1.8)	2.8 (0.4-3.8)	0.004
Upslope (%/minute)	185 (143–217)	141 (75–183)	0.016
AUC StO_2_	14.5 (11.2-21.3)	7.9 (4.3-12.6)	0.001
Tissue haemoglobin index	11.0 (9.1-12.8)	11.4 (9.5-13.7)	0.432

Data are presented as median (1st-3rd quartile). *StO*_*2*_ tissue O_2_ saturation, *AUC StO*_*2*_ area under the curve of reactive hyperaemia

### StO_2_, THI and outcome

**Fig. 1:**
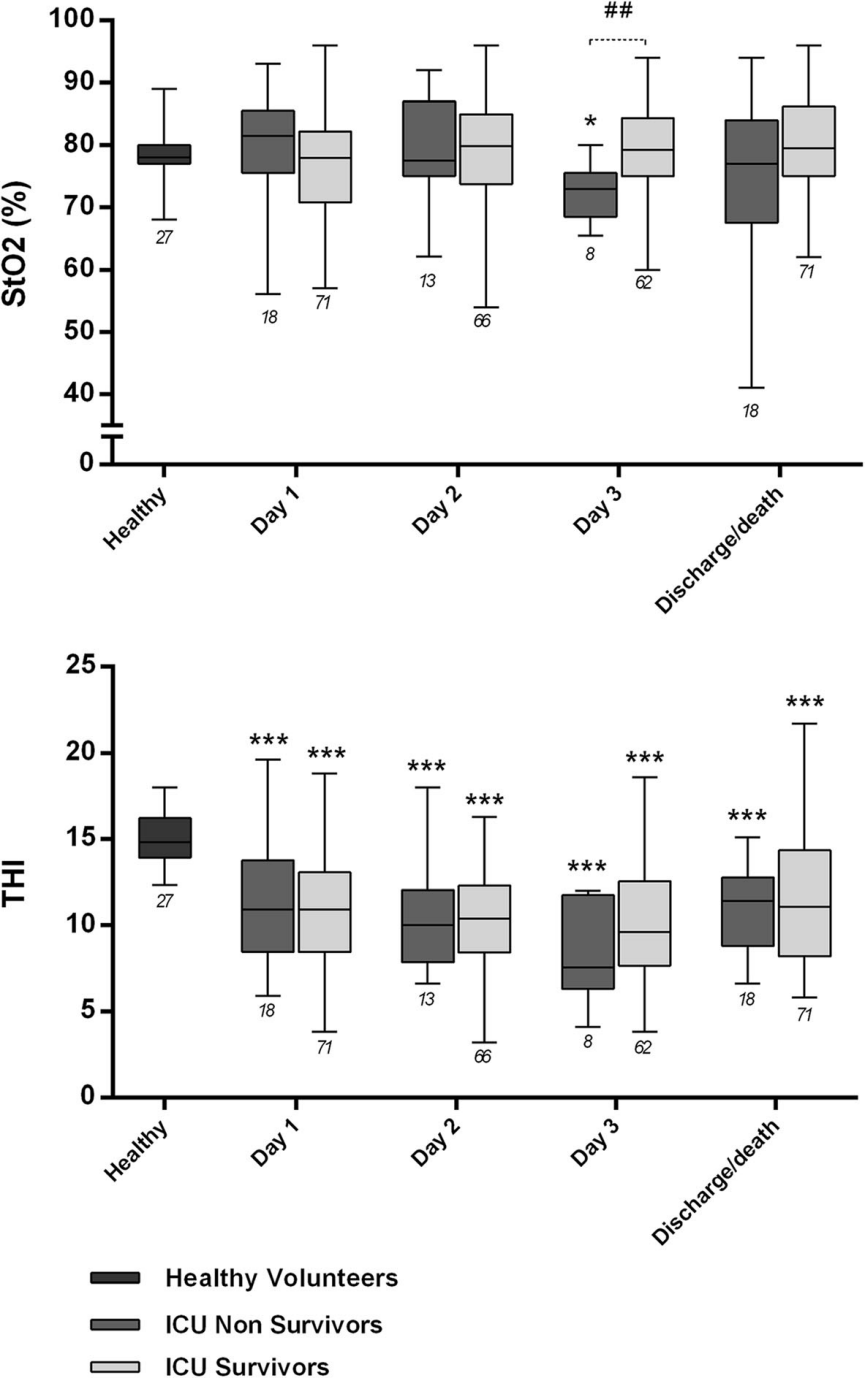
Tissue O_2_ saturation (*StO*_*2*_) and tissue hemoglobin index (*THI*) in healthy volunteers, ICU survivors and ICU non-survivors (on the first 3 days and on the day of death/discharge). *p < 0.05, **p < 0.01, ***p < 0.001, versus healthy volunteers, Kruskal-Wallis test with Dunn’s test for multiple comparisons. ^##^ *p* < 0.01, Mann–Whitney *U* test. Number of patients is indicated below the *error bars*

StO_2_ did not differ between healthy volunteers and patients on admission to the ICU (Fig. 1); however, ICU non-survivors had significantly lower values on day 3 as compared to healthy volunteers or ICU survivors. There were 16 patients (18 %) with StO_2_ < 70 % on admission to the ICU, but this was not associated with higher ICU mortality (12.5 % versus 23.5 % among those with StO2 ≥ 70 %, *p* = 0.274) or 90-day mortality (31 % versus 34 %, *p* = 0.498). Of 89 patients, 44 (49 %) had StO_2_ < 70 % at least once during their stay in the ICU, but this was not associated with worse outcome. Similarly, StO_2_ > 90 % (observed at least once in 38 patients) was not associated with higher ICU mortality or 90-day mortality. The THI was lower among patients as compared to healthy volunteers, with no difference between survivors and non-survivors (Table 2 and Fig. 1).

### Tissue O_2_ extraction rate and outcome

**Fig. 2:**
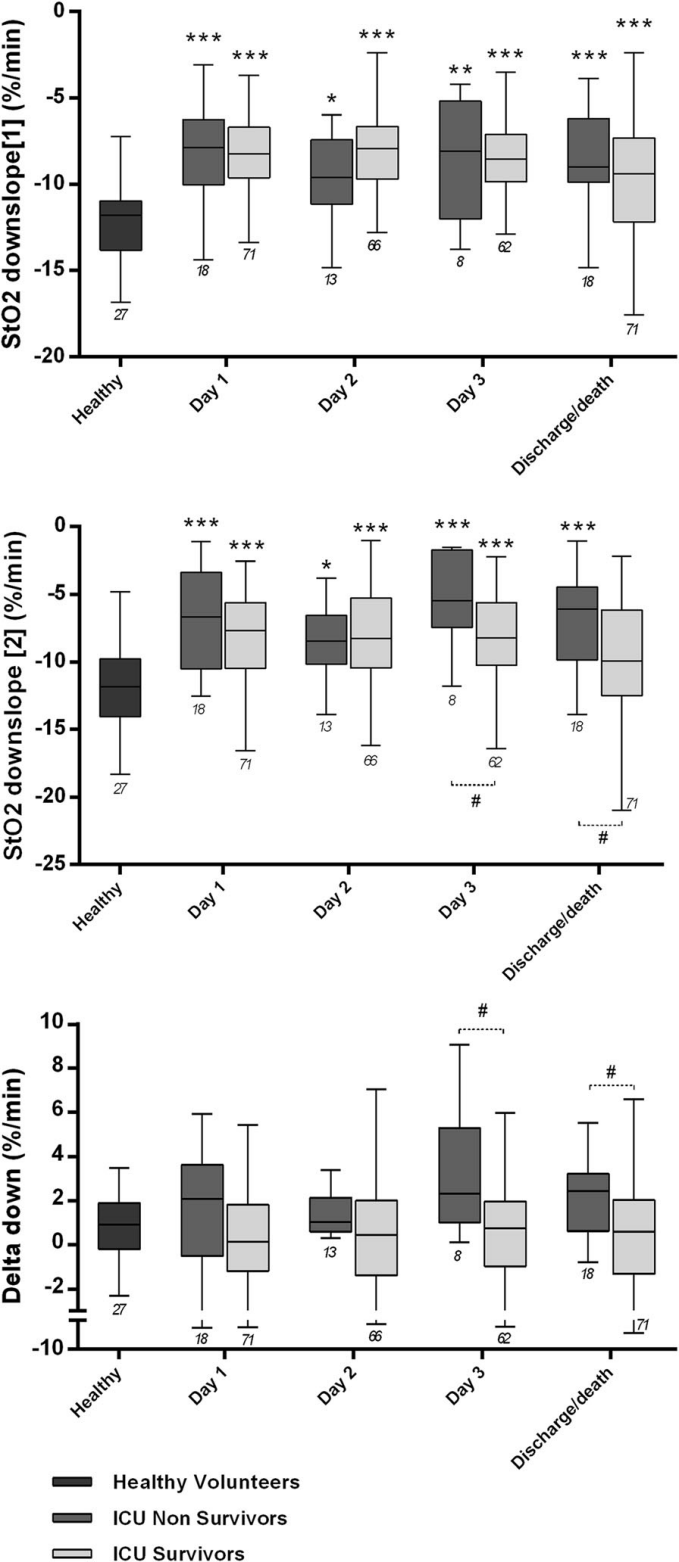
Downslope 1, downslope 2 and delta-downslope in healthy volunteers, ICU survivors and ICU non-survivors (on the first 3 days and on the day of death/discharge). *p < 0.05, **p < 0.01, ***p < 0.001, versus healthy volunteers, Kruskal-Wallis test with Dunn’s test for multiple comparisons. ^#^ *p* < 0.05, ^##^ *p* < 0.01, Mann–Whitney *U* test. Number of patients is indicated below the *error bars. StO*_*2*_ tissue O_2_ saturation

ICU non-survivors tended to have higher delta-downslope values (Fig. 2 and Table 2). While downslope 1 did not differ between survivors and non-survivors, a slower desaturation in the second phase of the ischemic challenge (higher downslope 2 and delta-dowslope) was observed among ICU non-survivors (Table 2). In pooled data, this was associated with sepsis, high lactate, hypotension or norepinephrine infusion (Additional file 1) and weak negative correlations were found between MAP and downslope 2 (*r* = −0.12, *p* < 0.001) or delta-downslope (*r* = −0.10, *p* = 0.004). Mean delta-downslope during the ICU stay discriminated 90-day non-survivors (area under the ROC curve = 0.72 (0.59, 0.84), *p* = 0.001). Patients in the fourth quartile of mean delta-downslope (>2.19 %/minute) had higher 90-day mortality (59 % in the fourth quartile versus 17 %, 23 % and 27 % in the first, second and third quartiles, respectively, *p* = 0.013). This association remained significant in Cox regression analysis after adjustment for APACHE II score on admission, MAP on admission and diagnosis of sepsis on admission or during the ICU stay (hazard ratio = 3.414 (95 % CI 1.084, 10.756), versus first quartile of mean delta-downslope, *p* = 0.036).

### Microvascular reactivity and outcome

**Fig. 3:**
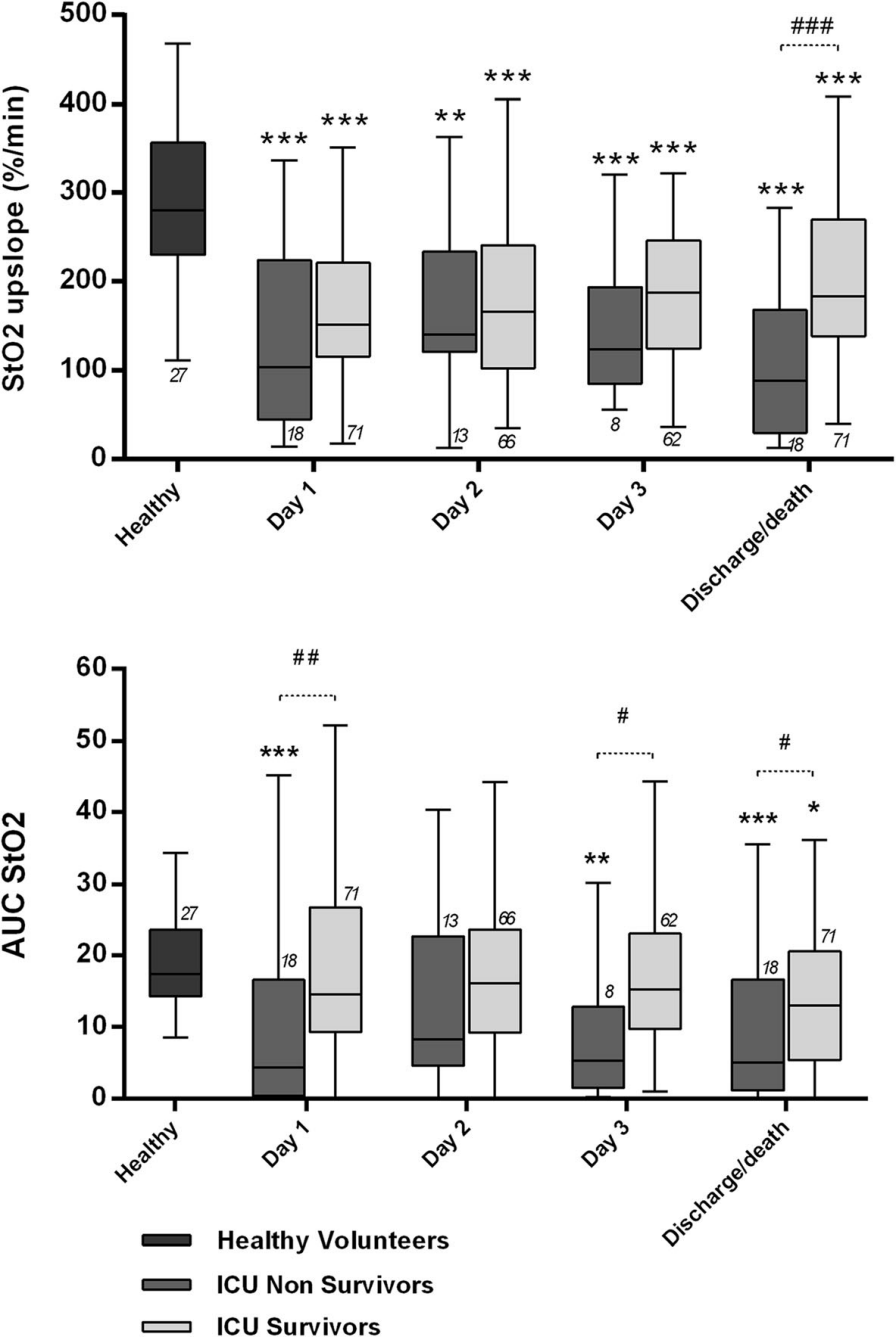
Upslope and area under the curve of the hyperaemic response (AUC StO_2_) in healthy volunteers, ICU survivors and ICU non-survivors (on the first 3 days and on the day of death/discharge). *p < 0.05, **p < 0.01, ***p < 0.001, versus healthy volunteers, Kruskal-Wallis test with Dunn’s test for multiple comparisons. ^#^ *p* < 0.05, ^##^ *p* < 0.01, Mann–Whitney *U* test. Number of patients is indicated below or above the *error bars. StO*_*2*_ tissue O_2_ saturation

The upslope was significantly lower in patients as compared to healthy volunteers, while AUC StO_2_ was altered only in ICU non-survivors (Fig. 3). There were more severe alterations in upslope and AUC StO_2_ among ICU non-survivors (Fig. 3 and Table 2). The presence of sepsis, high lactate, hypotension or norepinephrine infusion was associated with altered microvascular reactivity (Additional file 1). In pooled data, MAP was weakly correlated with the upslope (*r* = 0.18, *p* < 0.001) but not with the AUC StO_2_. The upslope on admission and its mean value during the ICU stay predicted 90-day survival (area under the ROC curve = 0.68 (0.54, 0.82) and 0.69 (0.57, 0.82) respectively, *p* < 0.01 in both cases). Similarly, the AUC StO_2_ on admission and its mean value predicted 90-day survival (area under the ROC curve = 0.70 (0.58, 0.82) and 0.68 (0.56, 0.80) respectively, *p* < 0.01 in both cases).

Patients in the lowest quartile of upslope on admission (upslope ≤88 %/minute) had higher risk for 90-day mortality (67 % versus 28 % for the fourth quartile, *p* = 0.004) independently of MAP and the presence of sepsis on admission or during the ICU stay; however, this association became non-significant when the APACHE II score was included in the model (hazard ratio = 2.607 (0.982, 6.919), *p* = 0.054). An AUC StO_2_ ≤ 6.65 (first quartile) on admission to the ICU was associated with higher risk of 90-day mortality, independently of the APACHE II score, MAP and the presence of sepsis on admission or during the ICU stay (hazard ratio 7.964 (2.211, 28.686) versus fourth quartile, *p* = 0.002).

### Prognostic value of NIRS-derived variables for mortality after ICU discharge

Among the 71 ICU survivors, 10 patients died within the first 90 days after ICU admission. During their stay in the ICU, these patients progressed to higher SOFA scores and lactate levels and lower upslope and THI values as compared to 90-day survivors, while no significant differences were found in the worst values of MAP, heart rate (HR), haemoglobin (Hb), partial pressure of oxygen (PaO_2_), central venous oxygen saturation (ScvO_2_) or the other NIRS-derived parameters (Additional file 2). The lowest upslope, lowest THI and lowest StO_2_ during the ICU stay were fair or good predictors of 90-day mortality, similar to the highest SOFA score or lactate levels (Additional file 3). A lowest upslope <68.8 %/minute predicted mortality after ICU discharge with 90 % sensitivity and 72 % specificity. In a multivariate logistic regression model including highest SOFA score, highest lactate, lowest MAP, highest HR, lowest ScvO_2_, lowest StO_2_, lowest upslope and lowest THI, only the lowest upslope was independently associated with survival after ICU discharge (odds ratio 0.970 (95 % CI 0.945, 0.996), *p* = 0.025).

## Discussion

Through daily NIRS monitoring of the skeletal muscle in conjunction with a VOT, we showed significant alterations in tissue Hb content, O_2_ extraction and microvascular reactivity in a heterogeneous population of critically ill patients in comparison to healthy volunteers. The introduction of an ischaemic challenge, as opposed to a static StO_2_ assessment, allowed us to evaluate the response of the tissue to a physiologic perturbation and its reserve capacity. While we did not find a clear and consistent relationship between StO_2_ or THI and outcome, an altered desaturation slope, slower re-oxygenation during reperfusion and less pronounced reactive hyperaemia were associated with mortality.

A number of studies indicate the prognostic value of skeletal muscle StO_2_ in several patient populations [7, 17–22]. In our study, the initial StO_2_ was similar to that observed among healthy volunteers in both ICU survivors and non-survivors, while a significant decrease was seen in ICU non-survivors only on day 3. We did not find any association between ICU mortality and mean StO_2_ during the ICU stay. Similar to the SvO_2_, StO_2_ reflects the balance between regional O_2_ delivery and consumption [6]. If on one hand a lower StO_2_ may result from a reduced O_2_ supply, an apparently stable or even higher StO_2_ may depend on a reduction in O_2_ extraction and consumption, which may be associated with worse outcome [23]. However, those patients who exhibited at least one StO_2_ < 70 % or >90 % during their ICU stay did not have a worse outcome. The muscle StO_2_ at rest could not have been sufficiently accurate to predict mortality in this population, in whom many factors (including global haemodynamics and oxygenation, use of vasopressors or sedation) may have influenced regional O_2_ levels and blood flow. A lower StO_2_ was, however, associated with sepsis, increased lactate or norepinephrine infusion, i.e. with conditions of likely hemodynamic instability, inadequate tissue oxygenation and activation of anaerobic metabolism.

The tissue O_2_ extraction rate was significantly reduced in critically ill patients as compared to healthy volunteers. More interestingly, while the first part of the downslope did not differ between survivors and non-survivors, the desaturation rate tended to be slower in the late ischaemic phase in non-survivors, and in sepsis, hypotension, high lactate levels or in patients receiving norepinephrine. A higher mean delta-downslope (i.e. a flattening in the second part of the desaturation curve) was independently associated with higher 90-day mortality, although a larger population would be needed to confirm the strength of this association.

To our knowledge, no other study has previously explored the potential relevance of variations in the desaturation slope during the ischaemic phase of the VOT. By using microelectrodes to measure muscle and subcutaneous oxygenation, Sair et al*.* showed that the decline in tissue O_2_ tension during ischaemia was initially more rapid in patients with sepsis as compared to controls, although the overall rate of decline was similar: this would suggest a reduction in the desaturation rate in the final part of the ischaemic challenge in these patients; however, the possible meaning of this was not discussed [24].

During ischaemia, the progressive decrease in local O_2_ levels triggers vasodilatory mechanisms including the release of adenosine triphosphate (ATP) from red blood cells or nitric oxide (NO) from S-nitrosylated-Hb [25]. In a model of cecal ligation and puncture in rats, Bateman et al. demonstrated a delayed capillary response time within hypoxic capillaries and an impaired release of ATP from erythrocytes in response to hypoxia, suggesting a loss of microvascular autoregulation [26]. We speculate that a constant tissue O_2_ extraction rate during ischaemia may reflect a more effective redistribution of blood flow to more hypoxic regions, facilitated by local microvascular vasodilation. A flattening in the final part of the desaturation curve may thus reflect altered microvascular autoregulation and limited tissue O_2_ extraction capacity in more severely affected patients.

The StO_2_ upslope and the AUC StO_2_ in the post-ischaemic hyperaemic phase are considered to reflect microvascular reactivity and endothelial integrity [6]. Previous studies show an association between a slower re-oxygenation rate and a worse outcome [27–29]. In this study the upslope did not significantly differ between ICU survivors and non-survivors during the first 3 days in the ICU, unlike the AUC StO_2_, which was higher on admission in survivors. Both the upslope and the AUC StO_2_ on admission, and their mean values during the ICU stay, were weak predictors of 90-day mortality. Importantly, however, among patients who were discharged alive from the ICU, the lowest upslope during the ICU stay was a fairly good predictor of 90-day mortality. This result remained robust in multivariate regression analysis, and would suggest that those patients who experienced a more severe impairment in microvascular function can remain at higher risk of an adverse outcome even after stabilization and normalization of clinical parameters.

Our study has several limitations. First, we could not evaluate the relationship between NIRS-derived variables and outcome in different disease categories (e.g. sepsis) due to the small number of patients and/or deaths in each subgroup. Second, our study cannot demonstrate a direct causal relationship between altered tissue oxygenation or microvascular dysfunction and outcome. Several studies have identified correlation between macro-haemodynamic parameters and NIRS-derived variables [30, 31]. In accordance with previous findings, our study showed more severe alterations in NIRS-derived variables in the presence of hypotension. Even if we cannot exclude reduction in perfusion pressure as the primary cause of the impairment in tissue O_2_ extraction and microvascular reactivity, it is noteworthy that the association between delta-downslope, upslope, AUC StO_2_ and survival was independent of MAP. Interventional studies incorporating NIRS parameters among resuscitation targets are needed to demonstrate a causal relationship between improved tissue O_2_ extraction or microvascular reactivity and better outcome.

By evaluating 89 patients daily for 8 (4, 15) days per patient, we performed more than 800 NIRS sessions, thus creating probably one of the largest existing databases. Nonetheless, the sample size could have been too small to evaluate differences in mortality, thus some analyses may have been underpowered. Moreover, results of the analyses of mean values during the ICU stay may be partly biased by the smaller number of measurements available for ICU non-survivors as compared to survivors due to a shorter ICU length of stay, possibly leading to overestimation of the statistical significance in these comparisons. Last, we did not assess other parameters that can be derived as part of the VOT, such as the nirVO_2_ (an index of local O_2_ consumption) [32], which may have proved of important physiologic and prognostic value.

## Conclusions

Our study confirms the association between altered NIRS-derived measurements and mortality in critically ill patients and supports the usefulness of NIRS monitoring in conjunction with a VOT for risk stratification of ICU patients. Moreover, this is the first study to explore the potential relevance of variations in the desaturation slope during ischaemia. A decrease in the desaturation rate in the last part of the ischaemic phase of the VOT was associated with a worse outcome, and could suggest impaired microcirculatory autoregulation, which limits the tissue O_2_ extraction capacity.

## Additional files

Additional file 1: NIRS-derived variables stratified based on the presence of sepsis, hypotension, tachycardia, high lactate levels and norepinephrine administration. (DOC 41 kb) Additional file 2: Worst values of clinical and NIRS-derived parameters during the ICU stay: comparison between 90-day survivors and 90-day non-survivors. ICU non-survivors were excluded from this analysis. (DOC 35 kb) Additional file 3: Receiver operating characteristics (ROC) curve analysis for 90-day mortality after ICU discharge. (DOC 52 kb)

## Abbreviations

APACHE II: acute physiology and chronic evaluation score
ATP: adenosine triphosphate
AUC StO_2_: area under curve of the hyperaemic response
Hb: haemoglobin
HR: heart rate
ICU: intensive care unit
MAP: mean arterial pressure
NIRS: near-infrared spectroscopy
NO: nitric oxide
O_2_: oxygen
ROC: receiver operating characteristics
SOFA: sequential organ failure assessment
StO_2_: tissue oxygen saturation
SvO_2_: central venous oxygen saturation
THI: tissue haemoglobin index
VOT: vascular occlusion test
