# Supplementary material for: Near-infrared spectroscopy for assessing tissue oxygenation and microvascular reactivity in critically ill patients: a prospective observational study
Source: Crit Care. 2016 Oct 1;20:311. doi: 10.1186/s13054-016-1500-5 (PMC5045573; doi:10.1186/s13054-016-1500-5)
Supplement: Additional file 1: — NIRS-derived variables stratified based on the presence of sepsis, hypotension, tachycardia, high lactate levels and norepinephrine administration. (DOC 41 kb) [file 13054_2016_1500_MOESM1_ESM.doc]

**Additional File 1 – NIRS-derived variables stratified based on the presence of sepsis, hypotension, tachycardia, high lactate levels and norepinephrine administration.**

|  | **StO_2_ (%)** | **Downslope 1 (%/min)** | **Downslope 2 (%/min)** | **Delta Downslope (%/min)** | **Upslope (%/min)** | **AUC StO_2_** | **THI** |
| --- | --- | --- | --- | --- | --- | --- | --- |
| Sepsis |  |  |  |  |  |  |  |
| *no (725)* | 81 [75-86] | -8.9 [-11.2, -7.2] | -8.5 [-11.1, -6.1] | 0.5 [-1, 2.5] | 183 [121-248] | 13 [6-21] | 11 [8-13] |
| *yes (98)* | 79 [73-83]* | -8.7 [-10.8, -7.0] | -7.2 [-9.1, -4.3]*** | 2.1 [0.3-3.9]*** | 145 [95-227]** | 11 [5-19] | 10 [7-12] |
| MAP |  |  |  |  |  |  |  |
| *≥65 mmHg (770)* | 80 [75-86] | -8.9 [-11, -7.1] | -8.4 [-10.9, -6] | 0.5 [-0.9, 2.6] | 185 [122-249] | 13 [6-21] | 10 [8-13] |
| *<65 mmHg (53)* | 81 [73-85] | -8.6 [-11.8, -7.1] | -7.3 [-9.9, -5]*** | 1.4 [-0.2, 3.3]*** | 120 [78-179]*** | 11 [5-20] | 10 [8-12] |
| Heart rate |  |  |  |  |  |  |  |
| ≤*90 bpm (605)* | 81 [75-86] | -8.6 [-10.8, -7.1] | -8.3 [-10.8, -6] | 0.4 [-1.1, 2.4] | 183 [124-241] | 13 [7-21] | 11 [8-13] |
| *>90 bpm (218)* | 80 [74-86] | -9.4 [-12.6, -7.6] | -8.4 [-10.9, -5.6] | 1.3 [-0.6, 3.8] | 169 [103-255] | 11 [5-19]* | 10 [8-13] |
| Arterial lactate |  |  |  |  |  |  |  |
| *≤1.5 mmol/L (680)* | 81 [76-86] | -8.7 [-10.8, -7.1] | -8.5 [-11, -6.2] | 0.4 [-1.1, 2.3] | 186 [127-248] | 13 [7-22] | 11 [8-13] |
| *>1.5 mmol/L (144)* | 79 [72-85]** | -9.2 [-12.4, -6.8] | -7.3 [-9.7, -4.3]*** | 2.3 [0.1-4.2]*** | 125 [85-222]*** | 11 [5-19]* | 10 [8-12]* |
| Norepinephrine |  |  |  |  |  |  |  |
| *no (587)* | 81 [76-87] | -8.8 [-12.3, -7.2] | -8.5 [-11, -6.2] | 0.4 [-1.1, 2.4] | 187 [131-253] | 13 [7-22] | 11 [9-13] |
| *yes (236)* | 79 [73-84]*** | -8.9 [-10.7, -7.1] | -7.3 [-10.2, -5]*** | 1.4 [-0.1, 3.4]*** | 150 [97-223]*** | 12 [6-20] | 10 [7-12]*** |

Numbers of measurements are indicated in parenthesis. *StO_2_* tissue O_2_ saturation, *AUC StO_2_* area under the curve of reactive hyperaemia, *MAP* mean arterial pressure. *p<0.05, **p<0.01, ***p<0.001, Mann Whitney U test.
