# Supplementary material for: Near-infrared spectroscopy for assessing tissue oxygenation and microvascular reactivity in critically ill patients: a prospective observational study
Source: Crit Care. 2016 Oct 1;20:311. doi: 10.1186/s13054-016-1500-5 (PMC5045573; doi:10.1186/s13054-016-1500-5)
Supplement: Additional file 2: — Worst values of clinical and NIRS-derived parameters during the ICU stay: comparison between 90-day survivors and 90-day non-survivors. ICU non-survivors were excluded from this analysis. (DOC 35 kb) [file 13054_2016_1500_MOESM2_ESM.doc]

**Additional File 2 – Worst values of clinical and NIRS-derived parameters during the ICU stay: comparison between 90-day Survivors and 90-day Non-Survivors. ICU Non-Survivors were excluded from this analysis.**

|  | **90-day Survivors (n=61)** | **90-day Non-survivors (n=10)** | **p** |
| --- | --- | --- | --- |
| Highest SOFA score | 7 [4-9] | 10 [8-12] | 0.021 |
| Highest HR (bpm) | 101 [87-112] | 104 [86-127] | 0.435 |
| Lowest MAP (mmHg) | 72 [64-79] | 71 [56-83] | 0.675 |
| Lowest Hb (g/dL) | 9.0 [8.3-10.1] | 9.7 [7.8-10.3] | 0.785 |
| Lowest PaO_2_ (mmHg) | 82 [70-109] | 84 [79-92] | 0.984 |
| Highest Lactate (mmol/L) | 1.4 [1.0-1.9] | 1.7 [1.5-2.2] | 0.099 |
| Lowest ScvO_2_ (%) | 69 [64-73] | 67 [61-71] | 0.159 |
| Lowest StO_2_ (%) | 70 [65-75] | 66 [58-70] | 0.076 |
| Highest Downslope 1 (%/min) | -5.5 [-6.7, -4.7] | -6.3 [-7, -4.4] | 0.693 |
| Highest Downslope 2 (%/min) | -3.4 [-6.1, -2.6] | -2.9 [-4.9, -2.5] | 0.486 |
| Highest Delta-Downslope (%/min) | 3.7 [1.7-5.3] | 4.5 [2.3-16] | 0.312 |
| Lowest Upslope (%/min) | 90 [66-145] | 46 [31-68] | 0.001 |
| Lowest AUC StO_2_ | 3 [0-10] | 5 [1-6] | 0.697 |
| Lowest THI | 7 [6-10] | 5 [4-6] | 0.015 |

*SOFA* Sequential Organ Failure Assessment, *HR* heart rate, *MAP* mean arterial pressure, *Hb* haemoglobin, *PaO_2_* arterial O_2_ tension, *ScvO_2_* central venous O_2_ saturation, *StO_2_* tissue O_2_ saturation, *AUC StO_2_* area under the curve of reactive hyperaemia, *THI* tissue haemoglobin index
