# Supplementary material for: Near-infrared spectroscopy for assessing tissue oxygenation and microvascular reactivity in critically ill patients: a prospective observational study
Source: Crit Care. 2016 Oct 1;20:311. doi: 10.1186/s13054-016-1500-5 (PMC5045573; doi:10.1186/s13054-016-1500-5)
Supplement: Additional file 3: — Receiver operating characteristics (ROC) curve analysis for 90-day mortality after ICU discharge. (DOC 52 kb) [file 13054_2016_1500_MOESM3_ESM.doc]

**Additional File 3 – Receiver operating characteristics (ROC) curve analysis for 90-day mortality after ICU discharge.**


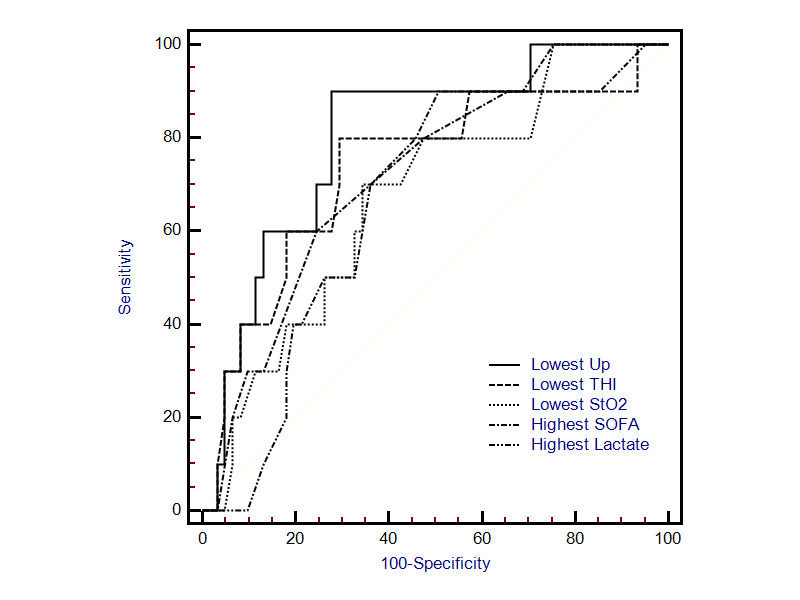


Lowest Upslope: area under the curve (AUC) 0.80 [95% confidence interval 0.69-0.89]; Lowest THI: AUC 0.74 [95% confidence interval 0.62-0.83]; Lowest StO_2_: AUC 0.68 [95% confidence interval 0.55-0.78]; Highest SOFA score: AUC 0.73 [95% confidence interval 0.61-0.82]; Highest Lactate: AUC 0.66 [95% confidence interval 0.54-0.77].
